# Managing ‘sick days’ in patients with chronic conditions: An exploration of patient and healthcare provider experiences

**DOI:** 10.1111/hex.13789

**Published:** 2023-06-08

**Authors:** Kirnvir K. Dhaliwal, Kaitlyn E. Watson, Nicole C. Lamont, Kelsea M. Drall, Maoliosa Donald, Matthew T. James, Sandra Robertshaw, Nancy Verdin, Eleanor Benterud, Kerry McBrien, Sarah Gil, Ross T. Tsuyuki, Neesh Pannu, David J. T. Campbell

**Affiliations:** ^1^ Department of Medicine, Cumming School of Medicine University of Calgary Calgary Alberta Canada; ^2^ Department of Medicine, EPICORE Centre University of Alberta Edmonton Alberta Canada; ^3^ Department of Medicine, Division of Nephrology University of Alberta Edmonton Alberta Canada; ^4^ Department of Medicine and Community Health Sciences, Cumming School of Medicine University of Calgary Calgary Alberta Canada; ^5^ Department of Family Medicine and Community Health Sciences, Cumming School of Medicine University of Calgary Calgary Alberta Canada; ^6^ Department of Medicine, Faculty of Medicine and Dentistry University of Alberta Edmonton Alberta Canada; ^7^ Department of Medicine, Community Health Sciences, and Cardiac Sciences, Cumming School of Medicine University of Calgary Calgary Alberta Canada

**Keywords:** chronic condition(s), qualitative research, sick day guidance, sick day management

## Abstract

**Introduction:**

People with chronic medical conditions often take medications that improve long‐term outcomes but which can be harmful during acute illness. Guidelines recommend that healthcare providers offer instructions to temporarily stop these medications when patients are sick (i.e., sick days). We describe the experiences of patients managing sick days and of healthcare providers providing sick day guidance to their patients.

**Methods:**

We undertook a qualitative descriptive study. We purposively sampled patients and healthcare providers from across Canada. Adult patients were eligible if they took at least two medications for diabetes, heart disease, high blood pressure and/or kidney disease. Healthcare providers were eligible if they were practising in a community setting with at least 1 year of experience. Data were collected using virtual focus groups and individual phone interviews conducted in English. Team members analyzed transcripts using conventional content analysis.

**Results:**

We interviewed 48 participants (20 patients and 28 healthcare providers). Most patients were between 50 and 64 years of age and identified their health status as ‘good’. Most healthcare providers were between 45 and 54 years of age and the majority practised as pharmacists in urban areas. We identified three overarching themes that summarize the experiences of patients and healthcare providers, largely suggesting a broad spectrum in approaches to managing sick days: *Individualized Communication, Tailored Sick Day Practices*, and *Variation in Knowledge of Sick Day Practices and Relevant Resources*.

**Conclusion:**

It is important to understand the perspectives of both patients and healthcare providers with respect to the management of sick days. This understanding can be used to improve care and outcomes for people living with chronic conditions during sick days.

**Patient or Public Contribution:**

Two patient partners were involved from proposal development to the dissemination of our findings, including manuscript development. Both patient partners took part in team meetings and contributed to team decision‐making. Patient partners also participated in data analysis by reviewing codes and theme development. Furthermore, patients living with various chronic conditions and healthcare providers participated in focus groups and individual interviews.

## 1 BACKGROUND

Approximately 44% of adults in Canada^1^ and 50% of adults in the United States^2^ have at least one chronic medical condition such as diabetes, chronic kidney disease, hypertension or heart disease. Chronic complications and progression of these conditions can be avoided by taking appropriate medications. Those living with these conditions are also susceptible to common acute illnesses like seasonal influenza,^3^ COVID‐19,^4^ and other gastrointestinal^5^ and pulmonary infections.^6^ Such illnesses can lead to volume depletion through fever, anorexia or fluid loss. When this occurs, some preventive medications can cause severe complications, such as acute kidney injury^7^ or hypoglycemia.^8^ These complications often require acute medical care,^7^, ^9^ and can be fatal.^8^, ^10^, ^11^ To prevent such complications, patients should temporarily adjust their medication routines during periods of acute illness, including temporarily withholding a number of medications, a strategy commonly referred to as ‘sick day management’. Several major international organizations (i.e., Diabetes Canada,^12^ the Canadian Cardiovascular Society,^13^ and the UK's National Health Service^14^) recommend that healthcare providers (HCPs) deliver this guidance to their patients.

We conducted a systematic scoping review on this topic and found minimal empiric evidence about the effectiveness of current approaches for the delivery of sick day guidance (SDG).^15^ Furthermore, only a few qualitative studies have reported on patients' and HCPs' experiences with interventions for delivering SDG like a ‘medicine sick day guidance’ card,^16^ and sick day rule plan.^17^ Two other studies have focused solely on patient experiences about self‐care with heart failure during sick days,^18^ and a telephone support intervention during sick days^19^ Qualitative studies are crucial to effectively capture behaviours, beliefs and experiences^20^—particularly to better understand a complex situation like navigating sick days and managing medications during periods of acute illness.

While a few interventional studies have been conducted in this area, a major gap remains in the literature as we still do not have a comprehensive understanding of the experiences of patients and HCPs with respect to SDG. We sought to comprehensively describe the experiences of patients managing sick days and of HCPs giving SDG to their patients.

## 2 METHODS

### 2.1 Design and framework

Qualitative description is a methodology that is well suited to provide a comprehensive summary of events and experiences.^21^ Researchers using this approach stay close to the data as compared to more interpretive qualitative methodologies.^21^ We undertook a qualitative descriptive study^21^, ^22^ to accomplish our study objective.

The theoretical domains framework (TDF) provided the conceptual underpinning to conceptualize the potential influences on the behaviours of interest in this study^23^ (i.e., patients managing sick days and HCPs providing SDG). We used the TDF to inform data collection and analysis, such as shaping our focus group, and interview guide questions, and codebook. Our study received ethical and operational approvals from research ethics boards at the University of Calgary (REB20‐1989) and the University of Alberta (Pro00103809).

### 2.2 Participants, sampling and recruitment

All study participants were required to be at least 18 years of age and be able to provide informed consent in English. The inclusion criteria for patient participants included:

1. taking two medications from the following classes: Renin–angiotensin–aldosterone system‐antagonists, diuretics, nonsteroidal anti‐inflammatory drugs (NSAIDs), sodium‐glucose transport protein 2 (SGLT2) inhibitors, insulin, sulfonylureas, meglitinides;
2. have access to an internet connection (i.e., able to attend online focus groups and complete electronic surveys) and/or a data‐enabled mobile device; and
3. able to read, write and speak English.

Patients were excluded if they had kidney failure which required chronic dialysis, were pregnant or they did not manage their own medications (i.e., this was done by a nurse or other professional).

The inclusion criteria for HCPs included:

1. practicing as a nurse practitioner (NP), pharmacist or primary care physician (PCP, i.e., family physician) in a community setting; and
2. possessed at least 1 year of experience working in this type of setting.

We undertook purposive sampling,^21^ aiming for maximum variation across domains such as age, sex and race (White and non‐White) for all participants, and type of chronic condition(s) for patients, and profession, practice area (rural vs. urban), and years of practice for HCPs.

Recruitment sources for patients included websites, university networks, specialist and PCP practice settings, community pharmacies, and other local, provincial and national organizations. PCPs and NPs were recruited through our contacts and other key stakeholders in the following clinical domains: nursing, primary care, endocrinology, nephrology and cardiology. Pharmacists were recruited through provincial organizations and personal connections.

### 2.3 Data collection

Data were collected using virtual focus groups (patients and pharmacists) and individual interviews (patients, NPs and PCPs) via Zoom™ teleconference software from January 2021 to April 2022. Focus groups were preferred, where possible, to enable participants to respond to each others' experiences, however, interviews were offered to those who were more comfortable with this format. K. M. D. developed the semi‐structured focus group and interview guides for patients and HCPs (Supporting Information: Table S1), which were reviewed by the team. The domains in the focus group and interview guides included patient experiences of acute illness and self‐care actions, patient knowledge about SDG, HCP provision of SDG, and HCP knowledge about SDG. Focus groups and interviews were audio recorded and team members (K. M. D., K. E. W. and N. C. L.) took field notes.

Three team members (K. M. D., M. D. and S. G.) and two patient partners (N. V. and S. R.) facilitated patient focus groups. Patients and care partners participated in 60‐min focus groups with approximately 6–8 people. The same three team members plus K. E. W. conducted the pharmacist focus groups. Pharmacists were invited to 60‐min focus groups with approximately 6–8 people. Another team member (K. K. D.) conducted the patient, NP, and PCP interviews. Each interview was approximately 60 min in duration. Data for all participant groups were collected until saturation was achieved—defined as no new emerging codes or findings in subsequent data collection.^24^

### 2.4 Data analysis

Audio recordings were transcribed verbatim by a professional transcriptionist. NVivo 12 (2018) and NVivo Release 1.0 (2020) software were accessed through the university institutions and used to manage transcripts, which were analyzed using qualitative conventional content analysis. Conventional content analysis is used ‘for the subjective interpretation of the content of text data through the systematic classification process of coding and identifying themes or patterns’.^25^ This is an appropriate analytic approach when there is a dearth of knowledge and research on a topic.^25^

**Figure 1:**
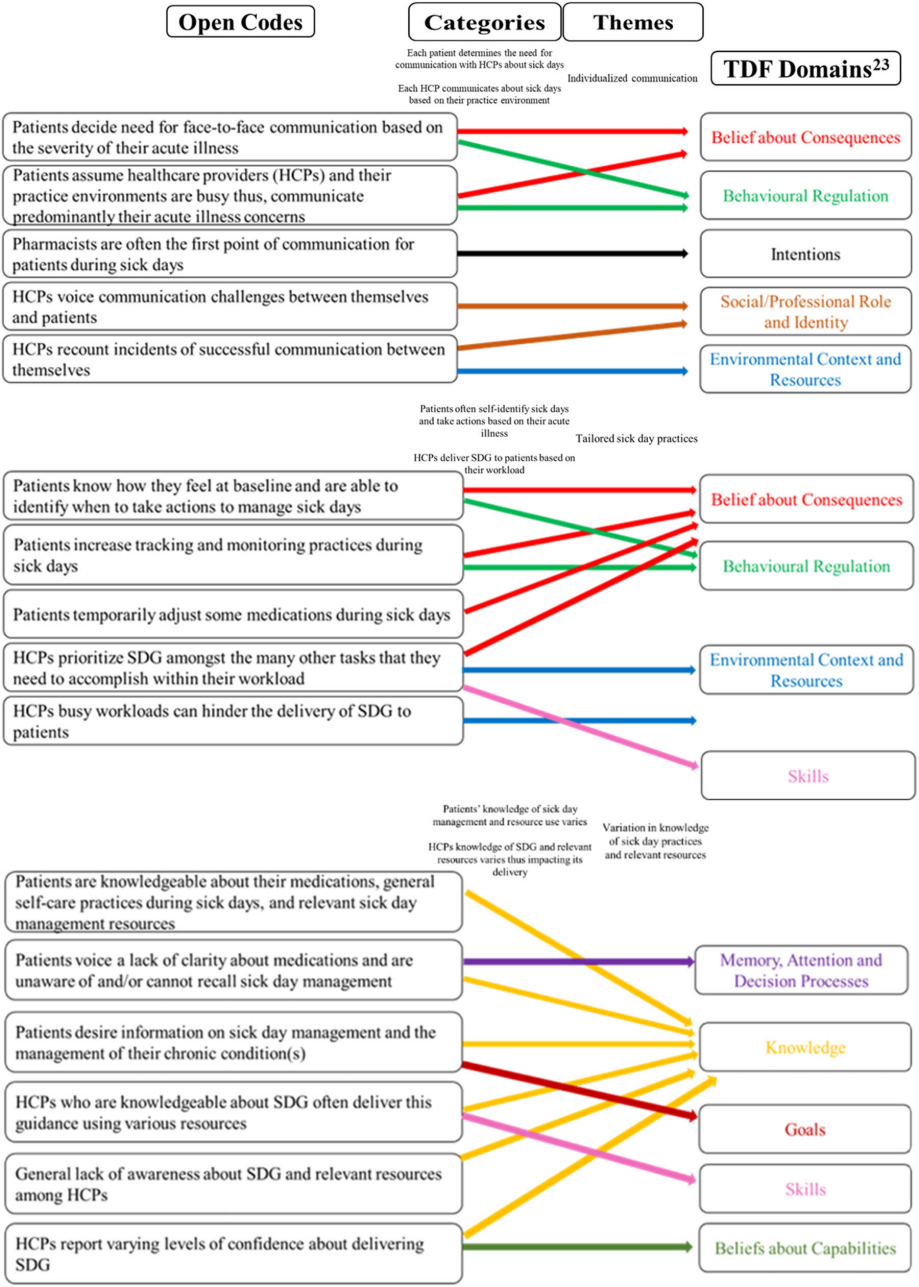
Findings relevant to theoretical domains framework (TDF).^23^ HCP, healthcare providers; SDG, sick day guidance.

Four team members (K. K. D., K. M. D., K. E. W. and N. C. L.) inductively analyzed each transcript independently. The following steps were taken to develop themes: each transcript was read; each transcript was reread to highlight interesting data; open codes were created from this data; the open codes were used to develop categories and then the main themes.^25^ We then reviewed our findings for relevance to the TDF, which illuminated the potential influences on patient management of sick days and HCP delivery of SDG (Figure 1).^23^ The team members regularly liaised with each other to discuss their work in progress and presented their emerging findings to the larger team and the senior investigator (D. J. T. C.), who provided guidance in the analytic processes.

### 2.5 Rigour

We used various strategies to enhance the trustworthiness of our findings such as triangulation, member checking, peer examination and maintaining an audit trail.^26^ Method triangulation was achieved by using both focus groups and individual interviews. Data triangulation was achieved by collecting data from different stakeholder groups (i.e., patients with varying chronic conditions, pharmacists, PCPs and NPs). Patient partners (N. V. and S. R.) aided in peer debriefing by providing feedback about the findings based on their lived experiences. We also conducted member checking by presenting a summary of the findings to pharmacists and seeking their feedback.

Our team is comprised of patients, HCPs, and research personnel who each contributed their personal expertize to the study. These varied perspectives were useful for conducting our study and bringing rich and nuanced interpretations to the data. One of the reasons we used such a large team was to ensure that no one individuals' experiences dominated the analysis and that the perspectives of patients and HCPs were both adequately represented while interpreting the data. We regularly engaged in reflexivity exercises where we reported on why we felt certain data had specific interpretations. We recorded these discussions and our reflections in a project diary, to maintain an audit trail. These practices (i.e., project diary, ongoing team meetings) were important to help team members understand how their beliefs, values and professional experiences impacted the work being conducted.^27^

## 3 RESULTS

Forty‐eight participants took part in this study (Tables 1 and 2). Patients participated in focus groups (*n* = 9) and individual interviews (*n* = 11). There was an even spread across the different patient age ranges. Pharmacists (*n* = 13) only participated in focus groups. PCPs (*n* = 12) and NPs (*n* = 3) took part in individual interviews. Most HCPs practiced in an urban area (*n* = 18).

**Table 1:** Self‐reported patient demographics.

Demographics Total number of patients in study (*n* = 20)	Number of patients
Age range	
<50 years	5
50–64 years	6
65–74 years	5
75 years and older	4
Marital status	
Single	3
Married	12
Divorced	1
Widowed	2
Common law	2
Education level	
Less than grade 12	1
College, trade school or university	18
Graduate school	1
Sex	
Female	10
Male	10
Other	–
Health status *2 participants missing	
Excellent	–
Very good	5
Good	10
Fair	3
Poor	–
Employment status	
Full‐time	8
Part‐time or casual	1
Not employed	–
Retired	11
Take medications for: (self‐reported, patients could select multiple options)	
Diabetes	15
Heart failure	4
Kidney disease	3
High blood pressure	16
I do not know	–
Place of residence	
Urban (population >500,000)	12
Rural (population <499,999)	8
Ethnicity	
Caucasian/White	17
Indigenous (First Nations/Inuit/Metis)	–
Visible minority	3

**Table 2:** Self‐reported healthcare provider (HCP) demographics.

Demographics Total number of HCPs in study (*n* = 28)	Number of HCPs
Type of HCP	
Nurse practitioner	3
Pharmacist	13
Primary care physician (family doctor)	12
Age range	
Under 35 years	5
35–44 years	8
45–54 years	10
55–65 years	5
Sex	
Female	18
Male	10
Practice area	
Urban (population ≥500,000)	18
Rural (population <499,999)	10
Years in practice	
Under 5 years	6
5–9 years	3
10–19 years	7
20–30 years	10
30 years and more	2

Our analysis revealed three overarching themes regarding patient experiences of managing sick days and HCP experiences of providing SDG to patients: *Individualized Communication, Tailored Sick Day Practices*, and *Variation in Knowledge of Sick Day Practices and Relevant Resources* (Table 3). We have also shown the potential affective, cognitive and environmental influences on patient management of sick days and HCPs delivery of SDG (Figure 1).^23^

**Table 3:** Thematic framework of findings

Open codes	Categories	Themes	Example quote
Patients decide need for face‐to‐face communication based on the severity of their acute illness.	Each patient determines the need for communication with HCPs about sick days.	Individualized communication.	‘I prefer face‐to‐face, yeah that's, especially if it's something really concerning…I mean like in person’. (Pt FG 3)
Patients assume healthcare providers (HCPs) and their practice environments are busy thus, communicate predominantly their acute illness concerns.			
			‘I mostly collaborate through fax unfortunately. I would love to be able to communicate more through phone with some physicians’. (Pharm FG 1)
Pharmacists are often the first point of communication for patients during sick days.			
HCPs voice communication challenges between themselves and patients.	Each HCP communicates about sick days based on their practice environment.		
HCPs recount incidents of successful communication between themselves.			
Patients know how they feel at baseline and are able to identify when to take actions to manage sick days.	Patients often self‐identify sick days and take actions based on their acute illness context.	Tailored sick day practices.	‘Most of the time it's just twice a day…If I'm feeling off then I will take it [blood sugar check] during the day’. (Pt Int 10)
Patients increase tracking and monitoring practices during sick days.			
			‘It [SDG] was a lot…more of a priority during the pandemic when they got actually sick with COVID’. (PCP Int 6)
Patients temporarily adjust some medications during sick days.			
HCPs prioritize SDG amongst the many other tasks that they need to accomplish within their workload.	HCPs deliver SDG to patients based on their workload.		
HCPs busy workloads can hinder the delivery of SDG to patients.			
Patients are knowledgeable about their medications, general self‐care practices during sick days, and relevant sick day management resources.	Patients' knowledge of sick day management and resource use varies.	Variation in knowledge of sick day practices and relevant resources.	‘Sometime for myself like if I have concern or like questions, I wonder things and I don't know who to ask’. (Pt FG 2)
Patients voice a lack of clarity about medications and are unaware of and/or cannot recall sick day management.			
			‘I use the Diabetes Canada handout for sick day management and I go through the handout with them’. (NP Int 2)
Patients desire information on sick day management and the management of their chronic condition(s).			
HCPs who are knowledgeable about SDG often deliver this guidance using various resources.	HCPs knowledge of SDG and relevant resources varies thus impacting its delivery.		
General lack of awareness about SDG and relevant resources among HCPs.			
HCPs report varying levels of confidence about delivering SDG.			

Abbreviation: SDG, sick day guidance.

### 3.1 Individualized communication

The theme of ‘individualized communication’ conveys that both patients and HCPs described how their individual circumstances determined the need and modality for communication about sick days (Table 3).

Many patients described assessing the severity of their acute illness to determine if and when to initiate communication with HCPs. Overall, many patients voiced intent to communicate with HCPs due to the fear of potential consequences (e.g., adverse medical complications) that might arise from not seeking professional assistance. Most patients attempted to self‐manage their sick days until they felt that it was not possible to continue on their own. Patients reported that they found it acceptable to receive virtual or telehealth advice for sick day self‐care but, when serious concerns arose, they preferred face‐to‐face communication with HCPs. For example, a patient reported: ‘I prefer face‐to‐face, yeah that's, especially if it's something really concerning…I mean like in person’ (Pt Focus Group [FG] 3). Whereas another patient stated:

*I manage things as well as I can…but if I were, on a Sunday things were not looking good or I had a concern, I feel that I could reach out to them [physician], but otherwise I will wait and if I think it's something serious I'll book [an] appointment with my family doctor*. (Pt FG 1)

Another factor that affected whether patients initiated contact with HCPs was the assumption that HCPs and their practice environments are extremely busy, so they often hesitated to communicate with providers unless absolutely necessary. One patient stated: ‘I mean it would be nice not to take up valuable time for the health professional’ (Pt FG 2).

Pharmacists were often the first point of communication for patients during sick days. For example, a patient‐reported communicating with a pharmacist about cold or flu medications: ‘Any time I have to, like I feel that I need to take something for a cold or a flu, I usually talk to my pharmacist’ (Pt Interview [Int] 2). Patients also communicated with pharmacists for medication‐related questions, blister packs, and general information about medications. One patient stated:

*I'm always kind of wondering what ones of these [over‐the‐counter] medications for this illness is it safe for me to even take? I mean you can read the boxes, but according to the boxes you can't take anything. So that's always a struggle for me. I never, you know, even when you get a cold, I mean that's where my pharmacist comes in handy*. (Pt Int 5)

Challenges in communicating about SDG were reported by all the different professions in our study. Some PCPs mentioned that they were not informed when other HCPs adjusted medications for sick days: ‘If they see another family doctor in another clinic or in a walk‐in, almost none of that [information] ever makes its way to us’ (PCP Int 12). The communication loop among pharmacists, physicians and patients was often described as being broken, and information was not shared or only provided one way. For example, a pharmacist stated that they relay patient information to physicians, who may not follow up with those particular patients: ‘The physician doesn't contact the patient, so again the consistency level of what we should expect when we are sending stuff to physicians is definitely not there’ (Pharm FG 1). Likewise, a NP reported experiencing being cut out of communication loops: ‘We are seen as invisible and “oh we [other HCPs] don't talk to nurse practitioners and we only talk to physicians” and so no, I never get anything’ (NP Int 2).

In contrast, other situations were recounted when HCPs successfully relayed communication about patient sick days to other professionals. Fax was the most commonly used medium for communication between HCPs, though some used EMR messaging, email or telephone. A pharmacist voiced: ‘I mostly collaborate through fax unfortunately. I would love to be able to communicate more through phone with some physicians’ (Pharm FG 1). Some pharmacists reported instances of the ideal bidirectional information flow: ‘I've had other physicians that call me up as soon as they see my fax and we talk over the phone’ (Pharm FG 1). Sometimes, general medication changes made by patients or other HCPs were communicated back to the prescriber: ‘I have really good communication from specialists generally with medication changes’ (PCP Int 1).

### 3.2 Tailored sick day practices

The theme of ‘tailored sick day practices’ conveys that patients and HCPs tailor sick day practices depending upon their specific context (Table 3).

Patients often reported having the knowledge to self‐identify sick days based on their acute illness context such as, the signs and symptoms they were experiencing, and they subsequently took actions in response to these symptoms. The specific actions taken included tracking and monitoring health status and adjusting medications during sick days. Patients voiced knowing how they feel at baseline allowing them to identify signs, symptoms, and feelings which were different and would indicate an acute illness: ‘It's easy for me to know when something is bad or is wrong. I am, how do you say? “J'écoute mon corps” – I listen to my body. I have my sixth sense’ (Pt FG 3).

In contrast, others described lacking the ability to differentiate between medication side effects, symptoms of their chronic condition(s), and acute symptoms that would constitute a sick day and require specific management: ‘Sometimes you don't know if it's low blood sugars or whether you are feeling like you've got the flu’ (Pt FG 2).

Some patients explained that they increased the frequency of their tracking and monitoring practices during sick days: ‘Most of the time it's just twice a day…If I'm feeling off then I will take it [blood sugar check] during the day’ (Pt Interview 10).

Patients also described different approaches to managing their medications during sick days. Some reported temporarily stopping particular medications when sick: ‘I do stop some of my medications. The other one that I would stop would be the [SGLT2‐inhibitor] and the water pill’ (Pt FG 2). Whereas another patient relayed that they did not stop their medications: ‘I am on heart medications, but it isn't recommended that I stop those’ (Pt FG 2).

HCPs attempted to prioritize the delivery of SDG to patients amongst the many other tasks that they needed to accomplish within their workload. PCPs reported prioritizing SDG when new medications were being initiated, but also when patients presented with acute illness: ‘It [SDG] was a lot…more of a priority during the pandemic when they got actually sick with COVID’ (PCP Int 6). Likewise, an NP reported delivering SDG when patients were acutely sick: ‘I have [an] mnemonic…the SADMANS mnemonic…I think this mnemonic kind of just gives me direction and structure of what do I need to stop, what do I need to continue’ (NP Int 1).

In contrast, during regular practice, SDG was often a low priority. For example, a PCP reported: ‘It's not something that we regularly capture in family practice’ (PCP Int 4). Rarely, some HCPs stated that they periodically reminded patients about SDG when not acutely sick: ‘Sometimes I will have refresher conversations’ (PCP Int 12). A pharmacist reported their perception that many PCPs may not prioritize the delivery of SDG:

*I'm not necessarily 100% sure whether that [SDG] comes into play when a [general practitioner] is seeing somebody who is coming in complaining of diarrhea and nausea and vomiting that their first thought is that this person has diabetes or is hypertensive and needs to stop these medications. I think they look more acutely to say, you know, what might be causing them and how do I help control it or resolve it*. (Pharm FG 3)

HCPs busy workloads can hinder the delivery of SDG to patients. HCPs often experienced time‐related constraints and competing demands. For example, pharmacists stated, ‘Sometimes [it] does come down to the time that I can spend with that patient’ (Pharm FG 1) and ‘there's two or three or four of us and you can only spend so much time’ (Pharm FG 3). PCPs also voiced similar issues and several reported that visits with patients were too short to cover all topics. For example, patients with multiple comorbidities or higher medical complexity required more time and energy, making the delivery of SDG more challenging than when a patient is on only one medication: ‘I have people who can have ten active diagnoses and 20 different medications and it's just too complex’ (PCP Int 7). Therefore, tasks like SDG were difficult to cover in detail and some felt that this aspect of care may be better delegated to other HCPs that have more time to spend with patients: ‘This sick day guidance could be something that they [pharmacists] provide teaching for’ (PCP Int 10).

### 3.3 Variation in knowledge of sick day practices and relevant resources

The theme of ‘variation in knowledge of sick day practices and relevant resources’ conveys that there is a spectrum of knowledge and resource use about sick day practices among both patients and HCPs (Table 3).

Many patients were knowledgeable about why they were prescribed their medications: ‘I do know why I'm taking them because I often ask the doctor’ (Pt FG 2). Many patients also voiced being knowledgeable about general self‐care practices during sick days such as, staying hydrated, resting, and taking acetaminophen instead of NSAIDs to avoid harm to their kidneys. For example, a patient stated: ‘Just went to bed and drank lots of fluids’ (Pt Int 7). Other patients used the Diabetes Canada patient resource for sick day management: ‘I quickly looked at Diabetes Canada’ (Pt FG 1).

In contrast, there were other patients who were less clear about why they were taking particular medications and the concept of sick day management. A few patients reported they had never been told about sick day management or, they could not recall having receiving guidance about what they should do with their medications in these situations. As a result, these patients did not take any specific actions when acutely sick:

*My family has a pretty significant history, like hereditary wise that a lot of us have high blood pressure and/or diabetes. So, I find that kind of concerning that I don't think my family members know that either because I've never heard of this [sick day management]. Like when I started my medications that I'm on right now, I know my mom in particular told me things about like high blood pressure medication, like don't eat grapefruit, that can affect it or stuff like that, but no one ever told me that if you are sick that you should stop. Now that's brand‐new information to me*. (Pt Int 6)

Patients conveyed a desire for reliable education and information about sick days and their chronic condition(s), in general:

*I mean if there is [rationale] for not taking particular drugs when you are sick, yes, I think that would be something that would be, you know, good to discuss with your specialist or family doctor*. (Pt Int 1)

Some patients sought information from the internet while others were unclear about what and who to ask: ‘Sometime for myself like if I have concern or like questions, I wonder things and I don't know who to ask’ (Pt FG 2). Several patients also expressed an interest in the use of electronic health tools for self‐management.

Similar to patients, there was a spectrum in terms of HCPs knowledge of SDG and relevant resources, impacting its delivery. Knowledgeable HCPs described using several resources. The use of Diabetes Canada SDG guidelines and patient handouts were commonly cited. A pharmacist stated: ‘Diabetes Canada is always one that I do refer to and make sure people have information on that’ (Pharm FG 1). PCPs also mentioned using this resource along with the locally developed chronic kidney disease pathway: ‘I often use the printout from the CKD Pathway…I think there is sick day guidance on there’ (PCP Int 1). Likewise, an NP stated: ‘I use the Diabetes Canada handout for sick day management and I go through the handout with them’ (NP Int 2).

In contrast, some PCPs were unaware of SDG resources or, chose not to use them when providing SDG: ‘I don't know that I have one right now’ (PCP Int 4). Another PCP stated: ‘I don't use any resources. It's all verbal’ (PCP Int 6). Overall, HCPs also voiced that their levels of confidence about sick day knowledge and its delivery varied. For example, a PCP mentioned having little confidence about SDG as it is not a part of their routine practice: ‘I couldn't say that I confidently remember to bring it up every time’ (PCP Int 9). Whereas, a pharmacist voiced more confidence in having detailed SDG conversations with patients—more than just about a sick day medication: ‘It's about trying to also get across to patients not only this is a sick day med [medication], but what does that sick or illness in this context really mean’ (Pharm FG 1).

## 4 DISCUSSION

We identified three overarching themes that help us to better understand patient experiences managing sick days and HCP experiences providing SDG: *Individualized Communication, Tailored Sick Day Practices*, and *Variation in Knowledge of Sick Day Practices and Relevant Resources*. Our study is unique in that we have described the experiences of patients in managing sick days and of HCPs providing this guidance to their patients in standard routine practice—rather than focusing on perspectives related to specific interventions regarding SDG. Moreover, we also demonstrated the potential affective, cognitive and environmental influences on patient management of sick days and HCPs delivery of SDG (Figure 1).^23^ This insight into patient and HCP behaviours and practices with respect to SDG in real‐world settings could be used to facilitate the development of tailored interventions to improve sick day care. The findings of our study corroborate and expand upon what is already known about this topic.

With respect to the theme of ‘individualized communication’, patients and HCPs in another study also prioritized face‐to‐face communication about SDG.^16^ Other authors have also reported that patients experiencing sick days need timely communication with HCPs to avoid adverse events (e.g., emergency department admission).^18^ Similar to our findings, pharmacists and physicians in another study have also previously identified the lack of effective communication between one another.^28^ Some of our HCPs spoke of successful interprofessional communication about patient sick days and medication management. Likewise, rural family physicians in another study have also reported that their communication with urban specialist providers was generally positive.^29^

In relation to the theme of ‘tailored sick day practices’, another study including people living with heart failure also reported varying abilities to identify symptoms thus, leading to challenges with self‐care strategies, including medication management during sick days.^19^ Similar to our findings, HCPs in another study have reported experiencing challenges in determining which guidance should take priority when caring for patients with complex medical histories.^17^ Multidisciplinary HCPs in other studies have also identified that the additional work of delivering SDG can be a barrier to delivering this guidance.^16^, ^17^

With respect to the theme of ‘variation in knowledge of sick day practices and relevant resources’, similar to our findings, some HCPs in other studies have also reported that they lacked confidence in delivering SDG to patients.^16^, ^17^ Other HCPs have identified the need for more knowledge such as data and best practices regarding temporarily stopping certain medications during sick days.^16^

There are several strengths of this research. First, the engagement of multiple stakeholders (i.e., patients, NPs, PCPs and pharmacists) offered unique and diverse perspectives about this topic. Second, we comprehensively described patient experiences managing sick days and HCP experiences providing SDG using rigorous qualitative methods. Third, we have provided crucial knowledge that can aid initiatives for improving care and outcomes for people living with chronic conditions during sick days. However, there are also several limitations that need to be considered. We conducted this qualitative research in the Canadian context so, our findings may not be fully representative of all populations of patients and HCPs outside of Canada. Given that this study was qualitative in nature, we cannot comment on how common the various themes might be in the broader population, for example, what proportion of patients struggle to identify the symptoms of a sick day event. Future quantitative (survey‐based) research could help to better understand the relative frequency of the various perspectives heard in this study.

## 5 CONCLUSION

The patient and HCP perspectives that were described by participants in our study are paramount to understand and consider for improving sick day care and outcomes for people living with chronic conditions. With respect to the theme of ‘individualized communication’, HCPs could educate patients on when communication is necessary with providers during sick days to avoid adverse medical complications. HCPs could also enhance communication about SDG between each other with the goal of improving patient experience and health outcomes. In relation to the theme of ‘tailored sick day practices’, patients who effectively identify the signs and symptoms of acute illness may be better equipped to implement appropriate sick day management. Therefore, there is a need for enhanced patient education and awareness to help patients develop these skills more uniformly across the patient population. The ‘variation in knowledge of sick day practices and relevant resources’ theme highlights that there is a wide range of knowledge and resource use practices among both patients and HCPs with respect to sick days. Both groups may benefit from formal, structured education and supporting resources to aid in effective sick‐day practices.^30^ If all HCPs developed the knowledge and used the resources specified by some of those in our study, it is likely that patient care and outcomes would improve for all.

## AUTHOR CONTRIBUTIONS

Maoliosa Donald, Matthew T. James, Ross T. Tsuyuki, Neesh Pannu and David J. T. Campbell conceived the idea for this study. David J. T. Campbell provided supervision for this study. Eleanor Benterud assisted as a research coordinator. Kerry McBrien provided expertize from a primary care medicine perspective. Kirnvir K. Dhaliwal, Kaitlyn E. Watson, Nicole C. Lamont, Kelsea M. Drall, Maoliosa Donald and Sarah Gil undertook data collection (see Section 2). Kirnvir K. Dhaliwal, Kaitlyn E. Watson, Nicole C. Lamont and Kelsea M. Drall undertook data analysis (see Section 2). Both patient partners (Sandra Robertshaw, Nancy Verdin) were involved from proposal development to the dissemination of our findings, including manuscript development (see Abstract). Kirnvir K. Dhaliwal drafted the initial manuscript, to which all authors contributed. All authors were responsible for the decision to submit this manuscript for publication.

## CONFLICT OF INTEREST STATEMENT

Matthew T. James is the principal investigator for an investigator‐initiated research grant from Amgen Canada (outside the submitted work). Ross T. Tsuyuki is the consultant for Shoppers Drug Mart and Emergent Biosolutions and Investigator‐initiated research grants from Merck, Sanofi, AstraZeneca, and Pfizer. Other authors declare no conflict of interest.

## ETHICS STATEMENT

Ethics approval prohibits sharing of individual‐level data.

## Supporting information

Supporting information.

## Data Availability

Aggregate data can be made available upon reasonable request to the corresponding author.
