# Supplementary material for: Managing ‘sick days’ in patients with chronic conditions: An exploration of patient and healthcare provider experiences
Source: Health Expect. 2023 Jun 8;26(4):1746–56. doi: 10.1111/hex.13789 (PMC10349256; doi:10.1111/hex.13789)
Supplement: Supplementary file 1 — Supporting information. [file HEX-26--s001.docx]

**Supplement 1: Focus Group and Interview Guides Relevance to Theoretical Domains Framework (TDF)^23^**

| **Focus Group and Interview Guides** | **TDF Domains^23^** |
| --- | --- |
| **Patient** |  |
| Overarching question: Tell us about the last time you were sick with a cold or the flu. What actions did you take? What kinds of support would you need/like?   - Did you contact your healthcare team? If yes, who did you contact? - Did you make any changes to your medications (e.g., stop, restart)? - What would you consider is sick enough to contact or phone someone (e.g., physician, pharmacist, etc.)? What type of symptoms would you be experiencing in this situation? - How might your approach to this have changed since the pandemic began last March? | Environmental Context and Resources: *Any circumstance of a person's situation or environment that discourages or encourages the development of skills and abilities, independence, social competence, and adaptive behaviour*  Beliefs about Consequences: *Acceptance of the truth, reality, or validity about outcomes of a behaviour in a given situation* |
| Specific experience with sick day management: Have you heard about medication sick day management/guidance or received this kind of advice before? If yes, think back to time we previously discussed.   - Who provided this advice? - How/when did you receive this advice? (e.g., paper, phone, before you got sick or when you were sick) - What challenges did you experience during that time? - What helped you remember and use sick day guidance? - What do you think could be done to help patients remember and use sick day guidance when they are ill? - Did you understand why you needed to stop your medications? - If no, would you have wanted advice? What type of advice, by who, how? | Knowledge: *An awareness of the existence of something*  Environmental Context and Resources: *Any circumstance of a person's situation or environment that discourages or encourages the development of skills and abilities, independence, social competence, and adaptive behaviour*  Memory, Attention and Decision  Processes: *The ability to retain information, focus selectively on aspects of the environment and choose between two or more alternatives*  Belief about Capabilities: *Acceptance of the truth, reality, or validity about an ability, talent, or facility that a person can put to constructive use* |
| Symptom/decompensation monitoring: When patients get sick, it may be important to understand and monitor their symptoms (e.g., fever, vomiting/diarrhea, etc.) and/or blood sugars, blood pressure, etc. in order to provide advice to keep them healthy. Think about a time when you had to fill out a questionnaire asking about your health status (e.g., questions about your condition, your management of your condition, your symptoms, etc.). Have you ever completed something like this?  If yes, provide one example of a time when this occurred for you and what you liked or disliked about the questions.   - When you are feeling sick and have symptoms such as diarrhea, headache or fever, would you or someone that supports your care be willing to complete surveys about these symptoms? | Memory, Attention and Decision  Processes: *The ability to retain information, focus selectively on aspects of the environment and choose between two or more alternatives*  Belief about Capabilities: *Acceptance of the truth, reality, or validity about an ability, talent, or facility that a person can put to constructive use* |
| **Pharmacist** |  |
| Tell us about your experience in assisting patients in managing their diabetes and blood pressure medications during “sick days”, i.e., during periods of acute illness.   - Do you provide counselling on sick day guidance, and if so, what information do you provide and how? (written, verbal) - How has this been affected by the pandemic? | Skills: *An ability or proficiency acquired through practice*  Environmental Context and Resources: *Any circumstance of a person's situation or environment that discourages or encourages the development of skills and abilities, independence, social competence, and adaptive behaviour* |
| Tell us about involving other providers (within your practice or external).   - Do you consult with other providers (e.g., primary care physician, specialists)? How? | Social/Professional Role and Identity: *A coherent set of behaviours and*  *displayed personal qualities of an*  *individual in a social or work*  *setting* |
| What roles do you think other providers play in providing medication sick day guidance (family physicians, specialists)? What roles do you think pharmacists play? | Social/Professional Role and Identity: *A coherent set of behaviours and*  *displayed personal qualities of an*  *individual in a social or work*  *setting* |
| If you do or were to provide medication sick day guidance, what barriers do/would you experience in providing this advice to patients? Facilitators?   - What are some reasons you may not provide or struggle to provide patients with sick day guidance? - What resources or tools may help you in providing sick day guidance? | Environmental Context and Resources: *Any circumstance of a person's situation or environment that discourages or encourages the development of skills and abilities, independence, social competence, and adaptive behaviour*  Knowledge: *An awareness of the existence of something* |
| What resources or tools do you perceive may help your patients adhere to sick day guidance? | Knowledge: *An awareness of the existence of something* |
| Are you currently informed if your patients are instructed to temporarily discontinue certain medications during times of an acute illness? How about when the medication is restarted? Would you like to receive this information?   - If yes, how would you like to be informed? How frequently? - If no, why not? | Social/Professional Role and Identity: *A coherent set of behaviours and*  *displayed personal qualities of an*  *individual in a social or work*  *setting* |
| **Primary care physician and Nurse practitioner** |  |
| Tell us about your experience in providing patients with anticipatory guidance for the management of their diabetes and blood pressure medications during “sick days” (i.e., periods of acute illness).   - What information do you provide and how? (written, verbal) - What is your practice for discussing these issues with patients? - How has this been affected by the pandemic? | Skills: *An ability or proficiency acquired through practice*  Environmental Context and Resources: *Any circumstance of a person's situation or environment that discourages or encourages the development of skills and abilities, independence, social competence, and adaptive behaviour* |
| Tell us about your experience in assisting patients with the management of their medications when a “sick day” occurs.   - Would you usually see a patient when they are having an acute illness or afterwards at a regular appointment? - How do you generally find out that your patient had/has an acute illness? | Skills: *An ability or proficiency acquired through practice*  Knowledge: *An awareness of the existence of something* |
| Tell us about involving other providers (within your practice or external).   - Do you or your patients consult with other providers about sick day guidance and protocols? - What roles do you think other providers play in sick day management (pharmacist, specialist, nurses)? - Are you currently informed if your patients are instructed to temporarily discontinue certain medications during times of an acute illness (i.e., by a pharmacist or other HCP) or when they are restarted?   - If yes, how are you informed? How frequently?   - If no, why not?   - Would you like to receive this information? How? | Social/Professional Role and Identity: *A coherent set of behaviours and displayed personal qualities of an individual in a social or work setting* |
| If you do or were to provide medication sick day guidance, what barriers do/would you experience in providing this advice to patients?   - What may influence you to provide or not provide or struggle to provide patients with sick day guidance? | Environmental Context and Resources: *Any circumstance of a person's situation or environment that discourages or encourages the development of skills and abilities, independence, social competence, and adaptive behaviour* |
| If you do or were to provide medication sick day guidance, what facilitators do/would you experience in providing this advice to patients?   - What resources or tools may help you in providing sick day guidance? | Environmental Context and Resources: *Any circumstance of a person's situation or environment that discourages or encourages the development of skills and abilities, independence, social competence, and adaptive behaviour*  Knowledge: *An awareness of the existence of something* |
| What resources/tools may help YOUR patients adhere to sick day guidance? | Knowledge: *An awareness of the existence of something* |
